# The impact of invasive *Sinanodonta woodiana* (Bivalvia, Unionidae) and mussel macroparasites on the egg distribution of parasitic bitterling fish in host mussels

**DOI:** 10.1038/s41598-025-93717-8

**Published:** 2025-03-19

**Authors:** Dariusz Halabowski, Kacper Pyrzanowski, Grzegorz Zięba, Joanna Grabowska, Mirosław Przybylski, Carl Smith, Martin Reichard

**Affiliations:** 1 Department of Ecology and Vertebrate Zoology, Faculty of Biology and Environmental Protection, University of Lodz, Lodz, Poland; 2 Institute of Vertebrate Biology, Czech Academy of Science, Brno, Czech Republic; 3 Modelling and Computational Science, Lodz Centre for Analysis, University of Lodz, Lodz, Poland; 4 Department of Botany and Zoology, Faculty of Science, Masaryk University, Brno, Czech Republic

**Keywords:** Invasive species, Mussel parasitism, Host-parasite interactions, Host selection, Parasite facilitation, Freshwater mussel, Evolutionary ecology, Invasive species

## Abstract

Facilitative and competitive interactions among coexisting parasite species, as well as among alternative host species, produce considerable ecological and evolutionary responses to host-parasite relationships. Such effects can be illuminated by impacts of non-native species on relationships in local communities. We used the association between parasitic European bitterling fish (*Rhodeus amarus*) and unionid mussels (which host bitterling eggs in their gills) to test the effects of the invasive Chinese pond mussel (*Sinanodonta woodiana*) and the presence of non-bitterling mussel macroparasites on the pattern of host mussel use by the bitterling across 12 unionid mussel communities with the absence or presence of *S. woodiana* (and variation in duration of coexistence with local species). While all six European mussel species were used by the bitterling (with the prevalence of > 30% in *Unio* spp.), no *S. woodiana* individual was infected by the bitterling. The presence of *S. woodiana* did not affect bitterling eggs distribution in native mussels. Large native mussels hosted more bitterling. Infection by non-bitterling parasites, mostly water mites (prevalence 47%) and trematodes (25%), did not affect rates of bitterling parasitism. We discuss our results in the context of the rapid evolution of non-native species in their new range and its implication on mussel conservation.

**Supplementary Information:**

The online version contains supplementary material available at 10.1038/s41598-025-93717-8.

## Introduction

Host-parasite relationships are complex and parasite species typically coexist with other parasites in the same host. This leads to intricate interactions between coexisting parasite species, facilitative or competitive. Facilitation occurs when one parasite enhances the fitness of the other parasite, whereas competition involves adverse effects. These interactions are fundamental in determining the success of parasite transmission and host health^1–4^. Facilitation effect can involve host immune modulation, where one parasite species suppresses the host’s defence against itself, but such immunosuppression helps the other parasite species. For example, the nematode *Heligmosomoides polygyrus* facilitates the survival of *Nippostrongylus brasiliensis* in the house mouse host by inhibiting the host’s rejection ability^5^ and nematode infection of Cape buffalo modulates immune response leading to facilitation effect on bovine tuberculosis infection^6^.

Competitive parasite-parasite interactions often reduce the fitness of one or more coexisting parasite species^7^. These can involve direct resource competition, interference, or immune-mediated competition. Within-host competition is known to affect phenotypes and community structure of parasites and host^8,9^. Parasites can distinguish between infected and uninfected hosts and between different parasite loads, showing a strong preference for uninfected hosts or hosts with a low parasite load^9^. Higher parasite load can drive stronger host defences and the evolution of more virulent strains^10^, but also increases competition within the parasite community for limited host nutrients. The balance of facilitation and competition among parasites considerably affects host population dynamics. Parasites can reduce host density through mortality or sterility^9^, altering competitive dynamics within populations. In freshwater ecosystems, parasite densities are regulated by host abundance and intraspecific competition^11^. Understanding associations between coexisting parasite species is crucial for broader insights into ecological and evolutionary processes.

The introduction of non-native species may alter traditional patterns within host-parasite networks, disrupting established interactions and affecting species richness and community structure^12^. It leads to various outcomes in host-parasite relations such as spill-over, spill-back and dilution effect^13^. Non-native hosts can harbour parasites that spill over to native taxa, leading to cascading effects on native hosts^14,15^. Thus, new parasites can spread independently of their original hosts, leading to secondary invasions of co-introduced parasites^16^. Non-native hosts can also facilitate the transmission of native parasites, resulting in spill-back effects where the non-native host serves as a carrier^17^. Alternatively, non-native species may represent less suitable hosts for native parasites and reduce transmission to native hosts through a dilution effect^18^. Host and parasite traits are crucial in predicting the potential for cross-species host-parasite exchange, underlining the importance of understanding these dynamics^19^.

The relationship between European bitterling (*Rhodeus amarus*) and freshwater mussels represents an unusual parasitic reproductive strategy where bitterling lay their eggs in mussels’ gills. The developing embryos remain inside the mussel, competing for resources with the mussel itself, damaging their gills^20^ and reducing their fecundity^21^. The European bitterling uses all native European unionid species as suitable hosts^22,23^ as well as several exotic mussel species^24,25^. The introduction and spread of invasive Chinese pond mussel (*Sinanodonta woodiana*) in European waters provided European bitterling with a potentially new host. Although this mussel species is known to be a common host for Asian bitterlings in its native range^26^, it was not demonstrated as the host of European bitterling during the initial stage of the *S. woodiana* invasion. One population of *S. woodiana* (Włocławski Reservoir, Vistula basin, tested in 2003) was readily used by European bitterling for oviposition, but all eggs were rejected from mussel gills within the first few days. In contrast, another *S. woodiana *population (River Kyjovka, Danube basin, tested in 2012) has been avoided for oviposition, despite being perceived as a possible host and extensively examined by male and female European bitterling^27^. In a captive environment (outdoor aquaculture tubs) where European bitterling had access exclusively to *S. woodiana* mussels, there were few juvenile bitterling recruits at the end of the reproductive season (M. Reichard, unpublished data), indicating that embryos of European bitterling may sometimes successfully complete their development in *S. woodiana*. This prompted the current study to test whether rapid evolutionary change (15–20 generations) has facilitated the utilisation of *S. woodiana* by European bitterling.

The aim of this study was to investigate how the presence of the invasive mussel *Sinanodonta woodiana* and the occurrence of mussel macroparasites affect the pattern of host mussel use by the European bitterling and its reproductive success. The increased risk of egg rejection by *S. woodiana *can be decisive for the bitterling oviposition choice. Both the presence and utilization of the new host were predicted to impact the use of native mussels. The presence of previously deposited bitterling embryos and non-bitterling parasites reduces oxygen condition in mussel’s gills and its quality as a potential host^28^. Thus, we predicted that mussels infected by non-bitterling macroparasites should be used less often by the bitterling. Specifically, we tested following hypotheses: (1) *S. woodiana* can be used as a host by European bitterling, especially at sites with the longest association; (2) presence of *S. woodiana* affects the pattern of use of European mussels by the bitterling, (3) there is negative relationship between bitterling parasitism and presence of non-bitterling parasites.

## Materials and methods

### Study design

**Fig. 1:**
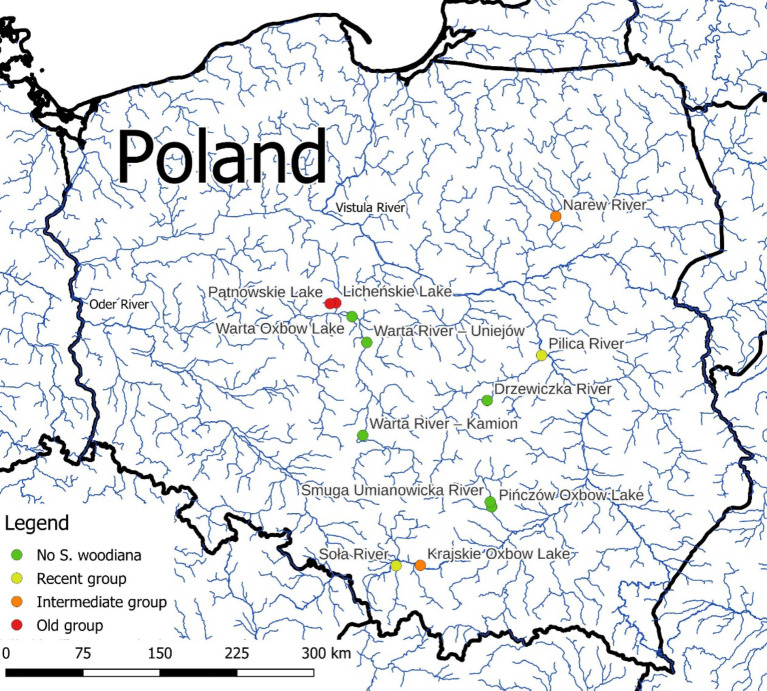
Study sites in relation to the treatment groups.

The study sites were selected on previously reported occurrence of bitterling and freshwater mussels^29^ and our pilot sampling one year before the study commenced. To determine the time of the first record of *Sinanodonta woodiana* at specific sites, we used an extensive database published in Mehler et al.^30^ and personal communication with A. M. Łabęcka (the source of Mehler et al. database). Selected sites were categorized into two main groups; (1) sites with *S. woodiana* present (sympatric) and (2) sites without *S. woodiana* (allopatric) in the mussel communities. The sympatric sites were further divided into three subgroups: the old-sympatry group (the first record of *S. woodiana* before 2000), the intermediate-sympatry group (the first record of *S. woodiana* between 2000 and 2015) and the recent-sympatry group (the first record of *S. woodiana* after 2015). Each site had stable populations of freshwater mussels, with communities including *Anodonta anatina*,* A. cygnea*,* Pseudanodonta complanata*,* Unio pictorum*,* U. tumidus*, and *U. crassus* s.l. Six bitterling-*S. woodiana* sympatry sites (two sites from each sympatry group) and 6 sites where *S. woodiana* did not occur (allopatric) were selected for the study (Fig. 1; Table 1). The research was conducted in June 2023 during the peak of the European bitterling reproductive season in Poland and following an early spring 2023 survey to confirm the presence of bitterling and mussel species. Mussels were collected by hand from the sediment when wading, snorkelling or diving in shallow water. We targeted to collect 25 mussel individuals of each species.

Bitterling presence and their relative abundance at mussel collection sites were assessed using electrofishing (EFGI 650, BSE Bretschneider Spezialelektronik, Chemnitz, Germany), personal communication, and data from the fish national monitoring program (Table 1).

### Mussel dissections

The collected mussels were sacrificed by cutting the abductor muscles. The shell length of each mussel individual was measured. Their gills, mantle, digestive system, and gonads were dissected under a stereoscopic microscope (Bresser Science ETD-201) and the number (and developmental stage) of each bitterling embryo and other parasites were recorded. All parasites were determined to the lowest possible taxonomic level and counted. We were interested in the relationship between bitterling and other taxonomic groups of non-bitterling parasites. We quantified the prevalence and abundance of water mites (parasitic nymphs and adults from the suborder Prostigmata), trematodes (parasitic worms of subclass Aspidogastrea), oligochaetes (parasitic species of Oligochaeta) and non-biting midges (parasitic species from the family Chironomidae).

### Data analysis

Across 12 sites, 1076 mussel individuals of seven species were collected. Only one mussel species was found at all sites (*Unio pictorum*) and two other species were common (*Anodonta anatina*,* U. tumidus*). Two mussel species were collected at a single site each (Table 1). Sampling was balanced with regard to the presence of non-native *Sinanodonta woodiana* (566 native mussels collected at sites where *S. woodiana* was present and 510 at sites where *S. woodiana* was absent).

All analyses were performed using the R statistical environment (v. 4.3.1)^31^. Statistical models were generated in the *glmmTMB *package^32^. Zero inflation, over- and under-dispersion of residuals, and model misspecification were checked using the *DHARMa *package^33^. Visualisation was performed in the *ggplot2 *package^34^. Prior to all formal statistical analysis, data were inspected for typographical errors, inconsistencies, outliers and covariance^35^. Data and R code for all analyses are available in the FigShare repository (10.6084/m9.figshare.23586384).

Bitterling load was initially expressed as three response variables, which were partly colinear but enabled responses to different questions. First, parasite prevalence (presence or absence of parasitism by respective taxon, Bernoulli distribution) was considered. Second, parasite abundance (mean abundance of bitterling eggs and embryos across all host mussels) was used to estimate the distribution of parasites across hosts. Third, bitterling clutch size was calculated as the number of bitterling offspring in an individual mussel (excluding mussels with no bitterling) to test how *S. woodiana*’s presence affected the distribution of bitterling eggs.

The role of the following factors was tested: mussel species (6 levels: native mussel species), presence of *S. woodiana* (present/absent) in the community, and abundance of trematodes and water mites in mussels (both as counts) as the main factors. Mussel size (measured as shell size along the longest axis, continuous, to the nearest 1 mm) and the presence of glochidia (binomial: present/absent) were used as covariates. The role of *S. woodiana* invasion history (4 levels: absent, recent, intermediate, old) was initially tested. However, the presence of *S. woodiana* was retained in the final models as this model had greater statistical power (and there was no difference in the outcome when the three levels of duration were considred separately). Four categories of *S. woodiana* invasion were retained for graphical outputs. The Collection site was modelled as a random intercept. *A. anatina* was set as the baseline species. *S. woodiana* was excluded from the set of mussels because (1) bitterling never used it, and (2) we tested the role of *S. woodiana*’s presence in the mussel community on the pattern of bitterling load among native mussels.

Given that two species, *U. tumidus* and *U. pictorum*, were used most frequently, the analysis was repeated for a subset consisting of these two species to confirm that the outcome has not resulted from the less frequent occurrence of certain mussel species. This more balanced design enabled us to corroborate the effects of covariates on bitterling load.

Bitterling prevalence was modelled as a Generalised Linear Mixed Model (GLMM) with Bernoulli distribution. Bitterling clutch size was analysed to test for the effects on the clustering of bitterling offspring. Linear Mixed Model (LMM) with log-transformed values provided a superior fit compared to GLMM with Poisson and truncated Poisson distributions (the fits were compared by the difference in Akaike Information Criterion, AIC). Again, the analysis was repeated for a subset containing only *U. tumidus* and *U. pictorum*.

**Table 1:** Characteristics of study design and research sites.

Group	Study site	River basin	Mussel species and their relative abundance	Bitterling relative abundance	Coordinates
*(a) Allopatric sites*					
No *S. woodiana*	Drzewiczka River	Vistula River	*A. anatina* (42%), *U. pictorum* (10%), *U. tumidus* (48%)	12%	N 51.45037, E 20.48654
No *S. woodiana*	Pińczów Oxbow Lake	Vistula River	*A. cygnea* (47%), *U. pictorum* (29%), *U. tumidus* (24%)	3%	N 50.517950, E 20.518673
No *S. woodiana*	Smuga Umianowicka River	Vistula River	*A. anatina* (10%), *A. cygnea* (42%), *U. pictorum* (25%), *U. tumidus* (23%)	19%	N 50.560955, E 20.499731
No *S. woodiana*	Warta Oxbow Lake	Oder River	*A. anatina* (24%), *A. cygnea* (34%), *U. pictorum* (19%), *U. tumidus* (22%)	6%	N 52.194623, E 18.581411
No *S. woodiana*	Warta River – Kamion	Oder River	*A. anatina* (15%), *U. crassus* s.l. (45%), *U. pitorum* (40%)	6%	N 51.153576, E 18.741061
No *S. woodiana*	Warta River – Uniejów	Oder River	*A. anatina* (18%), *Pseudanodonta complanata* (22%), *U. pictorum* (16%), *U. tumidus* (44%)	10%	N 51.968589, E 18.793521
*(b) Sympatric sites*					
Recent	Pilica River	Vistula River	*A. anatina* (22%), *A. cygnea* (2%), *S. woodiana* (12%), *U. pictorum* (35%), *U. tumidus* (29%)	8%	N 51.833734, E 21.270223
Recent	Soła River	Vistula River	*A. anatina* (26%), *S. woodiana* (42%), *U. pictorum* (32%)	5%	N 50.011145, E 19.200519
Intermediate	Krajskie Oxbow Lake	Vistula River	*A. anatina* (7%), *A. cygnea* (15%), *S. woodiana* (38%), *U. pictorum* (19%), *U. tumidus* (21%)	9%	N 50.012960, E 19.530882
Intermediate	Narew River	Vistula River	*A. anatina* (29%), *S. woodiana* (24%), *U. pictorum* (31%), *U. tumidus* (16%)	6%	N 53.047050, E 21.540356
Old	Licheńskie Lake	Oder River	*A. anatina* (19%), *S. woodiana* (38%), *U. pictorum* (29%), *U. tumidus* (14%)	9%	N 52.312995, E 18.349566
Old	Pątnowskie Lake	Oder River	*A. anatina* (32%), *S. woodiana* (24%), *U. pictorum* (16%), *U. tumidus* (28%)	7%	N 52.306674, E 18.266842

## Results

The overall prevalence of bitterling eggs and embryos was 18.8% (202 of 1076 mussels were parasitised), with major differences among mussel species, consistent across sampling sites (Table 2). Most notably, no bitterling offspring was found in *Sinanodonta woodiana* (*n* = 152), while bitterling eggs and embryos were found in all six native European mussels collected.

Non-bitterling parasites were detected in 59.9% of collected mussels. Water mites (prevalence 46.5%) were most common, followed by trematodes (24.9%), oligochaetes (3.1%) and nonbiting midges (1.6%). Water mites were the most common and reached the highest abundance in *Anodonta cygnea* (prevalence: 100%, n_total_ = 9083 individuals). Species- and site-specific prevalence of water mites and trematodes are shown in Supplementary Table 1.

**Table 2:** Prevalence and abundance of bitterling eggs and embryos across different mussel species and sites.

Group	Site	UP	UT	AA	AC	UC	PC	SW
W/out	Drzewiczka River	52 (25)	40 (25)	4 (25)	–	–	–	–
W/out	Pińczów Oxbow	8 (26)	12 (25)	–	0 (25)	–	–	–
W/out	Smuga Umian.	50 (24)	58 (24)	27 (11)	4 (25)	–	–	–
W/out	Warta – Kamion	36 (25)	–	4 (24)	–	8 (25)	–	–
W/out	Warta– Uniejów	18 (22)	41 (29)	4 (25)	–	–	4 (25)	–
W/out	Warta Oxbow	32 (25)	25 (24)	0 (26)	0 (25)	–	–	–
Rec	Pilica River	80 (25)	100 (24)	8 (25)	0 (2)	–	–	0 (25)
Rec	Soła River	68 (19)	–	13 (16)	–	–	–	0 (23)
Med	Krajskie Oxbow	25 (24)	12 (25)	10 (20)	0 (24)	–	–	0 (26)
Med	Narew River	23 (26)	5 (19)	4 (25)	–	–	–	0 (25)
Old	Licheńskie Lake	24 (25)	6 (16)	0 (25)	–	–	–	0 (26)
Old	Pątnowskie Lake	33 (21)	19 (26)	0 (27)	–	–	–	0 (27)
	Overall Prev	37 (287)	33 (237)	5 (249)	1 (101)	8 (25)	4 (25)	0 (152)
	Overall Abund	9.2 (1.0)	10.7 (1.1)	6.3 (2.8)	2.0 (10.1)	13.5 (7.2)	2.0 (10.1)	0
	N infected	106	79	13	1	2	1	0

The number of dissected mussels is given in parentheses.

W/out - group no *Sinanodonta woodiana*, Rec - recent group, Med - intermediate group, Old - old group, UP - *Unio pictorum*, UT* -* *U. tumidus*, AA - *Anodonta anatina*, AC - *A. cygnea*, UC - *U. crassus* s.l., PC - *Pseudanodonta complanata*, SW - *S. woodiana*.

### Bitterling prevalence

**Fig. 2:**
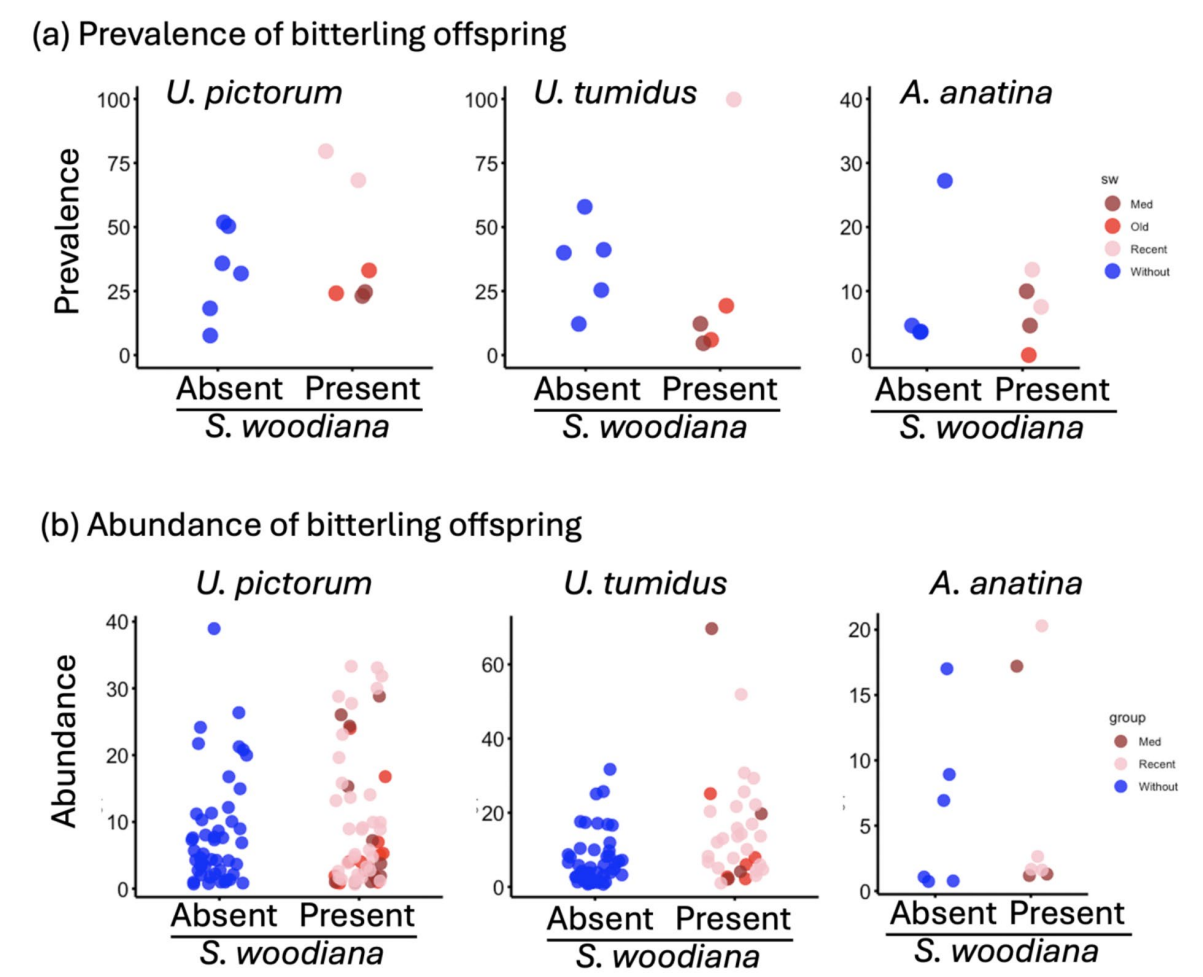
Prevalence (**a**) and abundance (**b**) of bitterling offspring across the three most utilised mussel species.

Host mussel species differed in the bitterling prevalence (Bernoulli GLMM on native host species: Table 3a, *n* = 923 native mussels), with *U. tumidus* and *U. pictorum* having higher prevalence than other host species (Table 3a: species-specific P-values are contrasts to the prevalence in *A. anatina*; Fig. 2). The presence of *S. woodiana* had no effect on distribution of bitterling eggs and embryos across native host mussels (*P* = 0.655). Likewise, mussel parasitism by trematodes (*P* = 0.155) and water mites (*P* = 0.544) nor the presence of mussel early developmental stages (glochidia) in their outer demibranchs (*P* = 0.401) had any effect on the presence of bitterling eggs and embryos in native mussels (Table 3a). Larger mussels tended to be used more often (*P* = 0.048). The analysis restricted to *U. tumidus* and *U. pictorum* (*n* = 524) fully corroborated outcomes from the full dataset (Supplementary Table 2a), with a strong positive role of mussel size on host use (*P* = 0.004).

### Bitterling clutch size

Bitterling clutch size (i.e., bitterling abundance in the subset of mussels which were infected) varied from 1 to 70 embryos and was significantly higher in *Unio* species (LMM on log-transformed data, *n* = 202, Table 3b: species-specific P-values are contrasts with the clutch size in *A. anatina*). There was no effect of *S. woodiana* presence of bitterling clutch size (*P* = 0.424) and no effect of trematode parasitism (*P* = 0.540), water mite parasitism (*P* = 0.265) nor mussel size (*P* = 0.174). However, the presence of glochidia negatively affected bitterling clutch size (*P* = 0.014). Fully concordant outcomes were obtained for the subset of *U. tumidus* and *U. pictorum* mussels (*n* = 185) (Supplementary Table 2b).

**Table 3:** Effects of host species, parasitism, and mussel size on Bitterling prevalence (a) and clutch size (b) in native mussels.

Coefficient	(a) Prevalence			(b) Clutch size			
	Est	95% CI	P	Est	95% CI	P	P
(Intercept)	− 5.12	− 7.11 to − 3.13	< 0.001	0.09	−1.28 to 1.46		0.900
Trematodes	− 0.08	− 0.18 to 0.03	0.155	− 0.02	− 0.10 to 0.05		0.540
Water mites	0.00	− 0.01 to 0.01	0.544	− 0.00	− 0.01 to 0.00		0.265
Mussel size	0.02	0.00 to 0.04	**0.048**	0.01	− 0.00 to 0.02		0.174
Glochidia [yes]	− 0.21	− 0.71 to 0.28	0.401	− 0.45	− 0.81 to − 0.09		**0.014**
Species [AC]	− 1.88	− 4.03 to 0.28	0.088	− 0.16	− 2.63 to 2.30		0.896
Species [PC]	0.45	− 1.73 to 2.63	0.684	− 0.86	− 2.98 to 1.26		0.424
Species [UC]	1.31	− 0.51 to 3.12	0.158	2.28	0.65 to 3.92		**0.007**
Species [UP]	3.01	2.26 to 3.76	**< 0.001**	0.78	0.17 to 1.39		**0.012**
Species [UT]	2.91	2.17 to 3.65	**< 0.001**	0.87	0.25 to 1.50		**0.006**
SW presence [Yes]	0.40	− 0.83 to 1.62	0.525	0.29	− 0.42 to 1.00		0.424
*Random effects*							
σ^2^	3.29			1.01			
τ_00 site_	1.00			0.28			
ICC	0.23			0.22			
N _site_	12			12			
Observations	923			202			
Marginal R^2^/ Conditional R^2^	0.370/0.517			0.087/0.286			

Statistically significant differences are in bold.

see Table 2.

## Discussion

### Differential host species use by the bitterling

No *Sinanodonta woodiana* mussel contained any bitterling egg or embryo, despite dissecting 152 individuals from 6 different sites, confirming that their resistance to host European bitterling is shared across populations, regardless of time since its population invasion and establishment. Two common native European mussel species, *Unio pictorum* and *U. tumidus*, were the most frequent host of bitterling eggs and embryos, with overall prevalence rates of 36.9% and 33.3%, respectively. Other native mussel species (*Unio crassus* s.l., *Pseudanodonta complanata*,* Anodonta anatina* and *A. cygnea*) were used less frequently, with an overall prevalence below 10%. The finding is in agreement with older reports from Central Europe^36,37^ and recent findings of Soler et al.^22^ and Marčić et al.^38^ that the European bitterling may utilise all coexisting European unionid species, but not the invasive *S. woodiana* of East Asian origin. In addition, data from various sites across Poland (River Odra and Vistula basins) demonstrate concordance in the species identity of the most commonly used host species – *U. pictorum* and *U. tumidus*. Those species are also most commonly used hosts at sites in the Danube basin (Czech Republic: Smith et al.^39^), while in England (where the bitterling is non-native), *U. pictorum* was common host while *U. tumidus* was used less often than *A. anatina*^40^. This inconsistency may be explained by the role of conditioning to the most common local species^41^. When comparing the mean number of bitterling eggs and embryos among different bivalve species, we found that *U. crassus* s.l. had the highest numbers. Although our study was limited to a single population of this species, Marčić et al.^38^ reported similar findings, where *U. crassus* was the third most frequently used host by bitterlings (very close to the number of bitterling eggs and embryos in *U. tumidus*). However, their study identified *A. anatina* as the species with the highest mean abundance of bitterling eggs and embryos. In contrast, our results showed higher mean abundance of bitterling offspring in *U. tumidus* and *U. pictorum*. In addition, we recorded the highest values for the maximum number of bitterling eggs and embryos in the gills of *U. tumidus*, which contrasts with the findings of Marčić et al.^38^. Their study reported the maximum number of bitterling eggs and embryos in the gills of *A. anatina*^38^, which is consistent with the study by Smith et al.^20^.

Despite two of the sampled sites being thermally polluted lakes (Licheńskie and Pątnowskie), the prevalence of bitterling eggs and embryos was similar to that observed in other studied standing waters. However, it cannot be ruled out that bitterling reproduction may be accelerated in thermally polluted environments, potentially leading to an underestimation of our results. This would, however, affect overall abundance of bitterling offspring in the mussels at the same rate of all species and should not bias our relative estimates of bitterling prevalence and abundance for each mussel species.

**Fig. 3:**
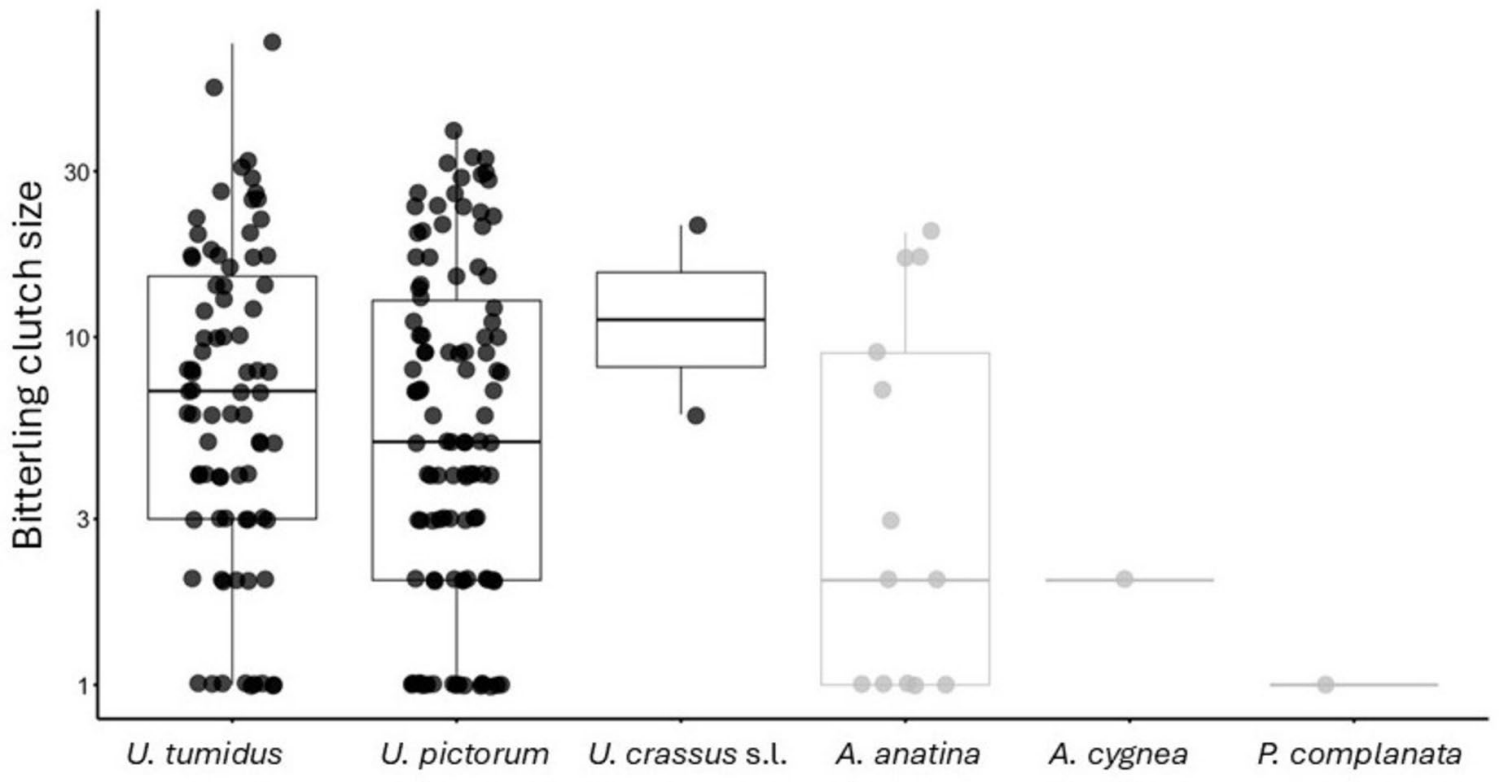
Bitterling clutch size (log-transformed) across different mussel species.

At the proximate level, bitterling choice of host mussels is related to the availability of dissolved oxygen for developing bitterling embryos, as it is a critical factor for embryo survival^28,42^. The *Unio* mussel species have significantly greater water filtration capabilities than *Anodonta *mussels^43^and may offer superior conditions to bitterling embryo development^44^. Accordingly, both the bitterling prevalence and clutch size were higher in *Unio* mussels (although bitterling prevalence but not abundance of *U. crassus* s.l. was relatively low, at least at a single site where it was present). The mussels from other genera were used much more sparsely (Table 2; Fig. 3).

Mussel choice is accomplished by active selection of the oviposition site by bitterling. Both male and female bitterling appear to perceive a gradient in oxygen concentration near the inhalant and exhalant siphons of potential hosts^28,42^ and use it to assess host ‘quality’^39^. Our results suggest that, within a natural range of shell size in our field sites, bitterling prefer to select larger mussels for oviposition. In addition, mussels which do not brood ripe glochidia contained more bitterling eggs and embryos, corroborating the experimental findings of Marčić et al.^38^ that bitterlings tend to actively choose host mussels without glochidia. This agrees with the hypothesis of female’s evaluation of “spatial occupation”^20,28,39,45^. Brian and Aldridge^46^ suggested that preference for older (rather than larger) mussels is more likely, given increased cumulative chance of parasite infection with age. The effects of size and age are difficult to disentangle, as they are strongly positively correlated in unionid mussels^47^ but given that bitterling spend approximately 3–5 weeks in the mussel gills, the cumulative effect of age does not appear relevant for bitterling parasitism. In conclusion, larger *Unio* individuals without glochidia in their outer demibranchs appear to be the most suitable bitterling hosts due to their optimal filtering capacity.

### The impact of ***S. woodiana*** on host use

*S. woodiana *was introduced to Europe in the 1970s^48^ and was well established in artificially heated lakes in Poland in 1993^49^. Its distribution has greatly expanded over the last 25 years across most of the European bitterling range^50,51^. *S. woodiana* is a common host of several Asian bitterling species, including *Rhodeus ocellatus*^26^, a generalist species related to the European bitterling^52^. While this could have led to the possibility that *S. woodiana*is commonly utilized by European bitterling as a suitable host, similar to other exotic mussel species^24,25^, this has not been demonstrated earlier^27,37^ or in the present study. There is often a lag phase before non-native species are included in local food webs and other community interactions^53,54^, associated with a rapid evolutionary change^55^ or learning to associate with a novel ecological partner^56^. We have shown that the period of up to at least 30 generations after introduction (for “old association treatment” *S. woodiana* populations) did not improve the ability of the European bitterling to use *S. woodiana* as a suitable host.

The fact that none of the 152 examined *S. woodiana* individuals from six different sites (with different periods since the introduction and hence association with the local European bitterling population) was infected by any bitterling egg or embryo agrees with recent findings of Marčić et al.^38^ from Croatia (River Sava basin). The failure of European bitterling to successfully utilize *S. woodiana* is most likely due to a persistent evolutionary lag between *S. woodiana* (which is well adapted to resist parasitism from previous long-time experience with multiple bitterling species in East Asia) and European bitterling (which is under a relaxed selection from evolutionarily naïve European unionid mussels)^36^. The proximate mechanism of this coevolutionary outcome is not known but may be related to *S. woodiana *capacity to expel bitterling eggs by sudden closure of the shell^36^, ability to decrease the level of dissolved oxygen concentration by extended shell closure (suffocating bitterling embryos) or differences in the size and anatomical structure of the gills between natural hosts of European bitterling (i.e. European unionids) and *S. woodiana*.

### Bitterling parasitism and the influence of non-bitterling parasites

Our third aim was to test whether non-bitterling parasites affected bitterling parasitism. Different groups of macroparasites interact inside their hosts^57^, such as a competition between oligochaetes and trematodes^58^. Parasite species can also facilitate each other’s presence in the host through modulation of the immune response^6^. We predicted competitive interactions between bitterling embryos and non-bitterling parasites in the mussel hosts, as it was observed among nematodes in mosquito larvae^9^, possibly with a geographic mosaic of interactions^7^. This prediction was based on a recent study on unionid mussel parasites which demonstrated that, in *A. anatina* mussels, bitterling parasitism was negatively associated with the parasitism by *Echinoparyphium recurvatum* trematodes – a parasitic worm of subclass Digenea, as well as with the presence of *Tetrahymena *sp. (Ciliophora)^46^.

Bitterling appear capable of detecting reduced host quality due to the presence of other parasites and preferentially oviposit in uninfected mussels^46^. In our study, however, we found no association with the prevalence or clutch size of the bitterling (Table 3, Supplementary Table 2) despite a high prevalence of bitterling and non-bitterling parasites. This suggests that, at least under the conditions studied, the presence of non-bitterling parasites in the mussel hosts does not interfere with bitterling reproduction. This is unexpected because it is well established that water mites cause physical damage to the mussel gills^59–61^. Damaged gills are supposed to be readily detected by the bitterling^62^ and negatively affect their decision to oviposit in such a mussel^28^. Trematode parasitism, also recorded at a high prevalence in our study, often reduces overall mussel condition. Trematode-infected mussels typically lack glycogen reserves and exhibit lower body weight^63,64^. However, in our study, we did not proceed with microscopic determination and did not determine trematodes at a lower taxonomic level. Therefore, our dataset may have lacked species which have more harmful effects on freshwater mussel condition^57,65^. On the other hand, this negative impact on mussel condition could perhaps be mitigated by the possibility of mussel castration^63^, as glochidia load had a measurable negative impact on bitterling parasitism in our study.

We had sufficient power to disentangle the outcome of potential competition between parasitic taxa. Non-bitterling parasites occurred in almost 60% of the examined mussels, with water mites being the most commonly recorded parasite taxon (Supplementary Table 1). We acknowledge that the determination of non-bitterling parasites to a precise taxonomic level was beyond the scope of our study and any possible species-specific effects could have been masked by our grouping of parasites to broader taxonomic categories. However, species-specific effects are especially plausible for positive interactions arising from immunomodulation^5,6^, while competitive interactions are most likely related to spatial effects and host resource use^8^. The lack of evidence of competitive interactions between bitterling and other parasites in the mussel gills and other internal tissues is unexpected.

One common ectoparasite of unionid mussels in Europe is zebra mussel (*Dreissena polymorpha*) and closely related dreissenid species^66^. *Dreissena* mussels attach to and impact freshwater mussels^21,46^ and decrease bitterling parasite load^67^. We have minimised the competitive effect of zebra mussels on the bitterling parasitism of unionid mussels in our study, as we specifically avoided sampling host mussels that were infected by non-native dreissenid bivalves, given that our sampling design primarily focused on comparing bitterling use of different unionid mussels and the role of internal non-bitterling parasites.

### Implications for mussel conservation and management of harmful species

The impact of parasitism on bivalves is poorly understood^68^. Unionid mussels are one of the most seriously threatened groups of Mollusca with a broad range of threats^69–71^, including competition with non-native *S. woodiana *and expansion of the bitterling^25,69–71^. *S. woodiana* possesses several characteristics contributing to its invasive success affecting native mussel populations and their conservation and management. They are year-round reproduction^72–74^, the lack of host-specificity for the hosts of glochidia^75^, the ability to outcompete native unionids for space and food^76–78^ and the development of cross-resistance in host fish causing a decrease in survival of native mussel glochidia^79^.

Although the presence of *S. woodiana *significantly reduces the reproductive success of the bitterling under experimental conditions^27^, our study indicates no significant differences in the prevalence and abundance of the bitterling in native mussel species in relation to the presence and absence of *S. woodiana*. Hence, even a high relative abundance of *S. woodiana* in the freshwater mussel community (30%) does not directly affect the reproductive success in natural populations of the bitterling. Thus, we did not observe any dilution effect in the European bitterling population.

Parasitic bitterling embryos constitute a significant cost to the reproductive success of the host mussels^21^. This prompts quantification of the potential impact of the bitterling on freshwater mussel populations and prevention of the invasion of bitterling into areas where it does not yet occur^23^. These efforts are particularly pressing, as the bitterling is often provided with legal protection based on its association with imperilled unionid mussels, while Van Damme et al.^80^ suggested that *Rhodeus amarus* is not a native species in much of its current range in Europe. While our study does not explicitly address the expansion dynamics of the bitterling, this perspective adds an interesting layer to the interpretation of its interactions with freshwater mussels. The potential consequences of its spread for native mussel populations warrant further investigation, particularly in regions where bitterling is expanding or where mussel populations are already under pressure.

## Conclusions

The overall patterns of bitterling parasitism across host mussel species corresponded with reports from previous studies. However, we did not find any evidence of trade-offs between bitterling and non-bitterling parasite prevalence. Given that negative associations between bitterling and non-bitterling parasitism of the unionid mussels were species-specific, future studies could benefit from a detailed taxonomic analysis of non-bitterling. Second, the use of *Sinanodonta woodiana *for offspring development in the bitterlings from East Asia but not in Europe calls for detailed research on the mechanisms of *S. woodiana* resistance to the European bitterling parasitism. Finally, although our results demonstrated no current threat of *S. woodiana* to the bitterling (as the abundance of native unionid mussels remains high), the long-term monitoring of bitterling and mussel populations is needed to assess long-term impacts of *S. woodiana* invasion and other environmental changes on these interactions.

## Electronic supplementary material

Below is the link to the electronic supplementary material.

Supplementary Material 1: Supplementary Table 1. Prevalence and abundance of water mites and trematodes across different mussel species and sites.

Supplementary Material 2: SupplementaryTable 2. Effects of host species, parasitism, and mussel size on bitterling prevalence (a) and clutch size (b) in the dataset of *U. tumidus* and *U. pictorum* (combined). Statistically significant differences are in bold typeset.

## Data Availability

All data generated or analysed during this study were uploaded to Fig Share repository (https://doi.org/10.6084/m9.figshare.23586384.v1).
