# Supplementary material for: The impact of invasive Sinanodonta woodiana (Bivalvia, Unionidae) and mussel macroparasites on the egg distribution of parasitic bitterling fish in host mussels
Source: Sci Rep. 2025 Mar 19;15:9417. doi: 10.1038/s41598-025-93717-8 (PMC11923366; doi:10.1038/s41598-025-93717-8)
Supplement: Supplementary file 1 — Supplementary Material 1: Supplementary Table 1. Prevalence and abundance of water mites and trematodes across different mussel species and sites. [file 41598_2025_93717_MOESM1_ESM.docx]

Supplementary Table 1. Prevalence and abundance of water mites and trematodes across different mussel species and sites

| **Site** | **UP** | **UP** | **UT** | **UT** | **AA** | **AA** | **AC** | **AC** | **UC** | **UC** | **PC** | **PC** | **SW** | **SW** |
| --- | --- | --- | --- | --- | --- | --- | --- | --- | --- | --- | --- | --- | --- | --- |
|  | **Water mites** | **Trematodes** | **Water mites** | **Trematodes** | **Water mites** | **Trematodes** | **Water mites** | **Trematodes** | **Water mites** | **Trematodes** | **Water mites** | **Trematodes** | **Water mites** | **Trematodes** |
| Drzewiczka River | 60 (25) | 4 (25) | 40 (25) | 36 (25) | 4 (25) | 16 (25) | – | – | – | – | – | – | – | – |
| Pińczów Oxbow | 100 (26) | 0 (26) | 100 (25) | 92 (25) | – | – | 100 (25) | 24 (25) | – | – | – | – | – | – |
| Smuga Umian. | 100 (24) | 0 (24) | 100 (24) | 0 (24) | 64 (11) | 0 (11) | 100 (25) | 0 (25) | – | – | – | – | – | – |
| Warta – Kamion | 48 (25) | 0 (25) | – | – | 29 (24) | 0 (24) | – | – | 4 (25) | 0 (25) | – | – | – | – |
| Warta– Uniejów | 9 (22) | 9 (22) | 0 (29) | 0 (29) | 4 (25) | 8 (25) | – | – | – | – | 4 (25) | 0 (25) | – | – |
| Warta Oxbow | 16 (25) | 0 (25) | 88 (24) | 38 (24) | 54 (26) | 27 (26) | 100 (25) | 40 (25) | – | – | – | – | – | – |
| Pilica River | 44 (25) | 36 (25) | 58 (24) | 46 (24) | 100 (25) | 60 (25) | 100 (2) | 100 (2) | – | – | – | – | 72 (25) | 0 (25) |
| Soła River | 32 (19) | 0 (19) | – | – | 69 (16) | 0 (16) | – | – | – | – | – | – | 13 (23) | 0 (23) |
| Krajskie Oxbow | 8 (24) | 33 (24) | 48 (25) | 64 (25) | 50 (20) | 45 (20) | 100 (24) | 33 (24) | – | – | – | – | 38 (26) | 0 (26) |
| Narew River | 12 (26) | 23 (26) | 0 (19) | 79 (19) | 5 (25) | 50 (25) | – | – | – | – | – | – | 8 (25) | 2 (25) |
| Licheńskie Lake | 84 (25) | 40 (25) | 6 (16) | 63 (16) | 100 (25) | 92 (25) | – | – | – | – | – | – | 42 (26) | 8 (26) |
| Pątnowskie Lake | 0 (21) | 0 (21) | 0 (26) | 62 (26) | 82 (27) | 70 (27) | – | – | – | – | – | – | 0 (27) | 4 (27) |
| Overall Prevalence | 44 (287) | 13 (287) | 43 (237) | 47 (237) | 48 (249) | 37 (249) | 100 (101) | 26 (101) | 4 (25) | 0 (25) | 4 (25) | 4 (25) | 29 (152) | 4 (152) |
| Mean Abundance | 7.62 | 0.28 | 12.92 | 1.92 | 13.82 | 1.15 | 89.93 | 0.52 | 0.04 | 0.00 | 0.04 | 0.00 | 2.30 | 0.09 |
| N of infected mussels | 126 | 36 | 102 | 111 | 117 | 89 | 101 | 26 | 1 | 0 | 1 | 0 | 44 | 6 |

Abbreviations: see Table 2
