# Supplementary material for: The impact of invasive Sinanodonta woodiana (Bivalvia, Unionidae) and mussel macroparasites on the egg distribution of parasitic bitterling fish in host mussels
Source: Sci Rep. 2025 Mar 19;15:9417. doi: 10.1038/s41598-025-93717-8 (PMC11923366; doi:10.1038/s41598-025-93717-8)
Supplement: Supplementary file 2 — Supplementary Material 2: SupplementaryTable 2. Effects of host species, parasitism, and mussel size on bitterling prevalence (a) and clutch size (b) in the dataset of U. tumidus and U. pictorum (combined). Statistically significant differences are in bold typeset. [file 41598_2025_93717_MOESM2_ESM.docx]

Supplementary Table 2. Effects of host species, parasitism, and mussel size on bitterling prevalence (a) and clutch size (b) in the dataset of *U. tumidus* and *U. pictorum* (combined). Statistically significant differences are in bold typeset

|  | **Prevalence** | | | **Clutch size** | | | |
| --- | --- | --- | --- | --- | --- | --- | --- |
| *Coefficient* | *Est* | *95% CI* | *P* | *Est* | *95% CI* | | *P* |
| (Intercept) | -3.23 | -5.26 – -1.19 | 0.002 | 0.47 | -0.84 – 1.78 | | 0.479 |
| Trematodes | -0.04 | -0.15 – 0.06 | 0.414 | -0.03 | -0.11 – 0.05 | | 0.510 |
| Water mites | 0.00 | -0.01 – 0.02 | 0.486 | -0.00 | -0.01 – 0.00 | | 0.401 |
| Mussel size | 0.03 | 0.01 – 0.05 | **0.004** | 0.01 | -0.00 – 0.03 | | 0.059 |
| Glochidia [yes] | -0.31 | -0.83 – 0.22 | 0.250 | -0.45 | -0.81 – -0.09 | | **0.014** |
| Mussel species [UT] | -0.20 | -0.66 – 0.27 | 0.411 | 0.08 | -0.26 – 0.42 | | 0.641 |
| SW presence [Yes] | 0.61 | -0.81 – 2.03 | 0.399 | 0.37 | -0.33 – 1.07 | | 0.303 |
| **Random Effects** | | | |  |  | |  |
| σ^2^ | 3.29 | | | 0.99 | |  |  |
| τ_00_ _site_ | 1.37 | | | 0.26 | |  |  |
| ICC | 0.29 | | | 0.21 | |  |  |
| N _site_ | 12 | | | 12 | |  |  |
| Observations | 524 | | | 185 | |  |  |
| Marginal R^2^ / Conditional R^2^ | 0.041 / 0.323 | | | 0.059 / 0.254 | |  |  |

Abbreviations: see Table 2
